# Notes and News

**Published:** 1906-01-27

**Authors:** 


### NOTES AND NEWS.

THE Evelina Hospital for Sick Children is making a public appeal for £10,000 to defray the cost of building a new out-patient department.

ACCORDING to a recently published official statement, tetanus is extremely prevalent in Cuba, especially among infants. During the last five years over 25 per 1,000 of new-born infants have died of tetanus.

MR. ALEXANDER LAING, who died last October, has bequeathed £1,000 to the Royal Infirmary, Newcastle-upon-Tyne, for the purpose of maintaining a bed or cot in memory of his late wife, and £300 each to numerous other hospitals and charitable institutions.

LAST week a chemist was summoned at the Marylebone Police Court for selling an impure product to a customer who had asked for a pint of distilled water. An official analysis of a sample of the water showed it to be very dirty and full of mouldy growths, and it left an appreciable deposit on evaporation. In the defence it was urged, in the first place, that the chemist thought the article was to be used for the purposes of photography, and, secondly, that water was not a drug within the definition of the Food and Drugs Act. The magistrate did not accept either of these pleas, and imposed a fine of 10s., with £3 3s. costs.

H. M. S. *Black Prince*, which is now ready to be commissioned and is the latest and most powerful type of armoured cruiser, will be on view in the Victoria Docks, at a small charge, on January 27 and February 3 and 4 in the afternoons. The proceeds will be given to the Seamen's Hospital, Greenwich, the Poplar Hospital, and the West Ham Hospital. It is to be hoped that the kindness of the Thames Ironworks Shipbuilding and Engineering Company and of the Admiralty in permitting this public inspection will be much appreciated by the public, who will be thus enabled at the same time to benefit both themselves and three most admirable and deserving charities.

SIR R. DOUGLAS POWELL, BART., has consented to preside at the annual meeting of the After Care Association for Poor Persons discharged recovered from Asylums for the Insane to be held at his house on February 15 next at 3 P.M.

MR. WILLIAM ROBERT LANE, of Richmond, Surrey, who died last November, has left £1,000 each to the Queen's Hospital, Birmingham, the Birmingham General Hospital, and the Birmingham and Midland Free Hospital for Sick Children.

IN accordance with their annual custom, the Poplar Guardians have voted various sums of money to charitable institutions which aid in the work of the Board. Grants have been made to London Hospital, Poplar Hospital, Victoria Park Consumption Hospital, and the Surgical Aid Society.

IN the Probate Division this week a case was mentioned which had reference to the will of the late Mr. John R. Davidson, who died in 1902, leaving the bulk of his property, £48,000, to St. Thomas's Hospital. The defendant, a sister of the testator, was, said counsel, a lunatic, and the hospital authorities had agreed that £10,000 should be set aside for the support of her relatives. The will was thus formally proved and pronounced for.

IN the course of a lecture on "Military Hygiene on Active Service," given at the Royal United Service Institution last week, Major T. H. J. C. Goodwin, D.S.O., made a strong appeal for improved sanitation in the Army. He pointed out, as many others before him have pointed out, that in our recent campaigns the losses from wounds, as compared with the losses from preventible disease, have been very small; and he referred to the 41,000 cases of enteric fever among our soldiers during the Boer War to enforce his argument. With this deplorable breakdown he contrasted the relative immunity from disease of the Japanese soldiers in Manchuria —a country where intestinal diseases might well have been expected to cause havoc in a belligerent army. As a matter of fact the Japanese lost only 15,000 men from sickness, as compared with 57,000 from wounds, a proportion without parallel in the records of war. Major Goodwin thought two reforms must be established if we are to emulate the good example set by the Japanese. The first is sanitary organisation, and the second is the further education in sanitary principles of regimental officers and men. The latter appears to us to offer the only solution of the problem. Unless the combatant members of the Army can be taught to understand the reason of the only preventive measures at present available, little or no improvement is to be expected. The objection that the requisite reforms would entail additions to the non-combatant portion of an army is beside the mark when the great reduction by sickness of the fighting strength is considered. To achieve the results which the Japanese have gained at a loss of 15,000 soldiers from disease, the British Army under similar circumstances would probably have lost a quarter of a million men.

